# Skill Training Periodization in “Specialist” Sports Coaching—An Introduction of the “PoST” Framework for Skill Development

**DOI:** 10.3389/fspor.2019.00061

**Published:** 2019-11-15

**Authors:** Fabian W. Otte, Sarah-Kate Millar, Stefanie Klatt

**Affiliations:** ^1^Department of Cognitive and Team/Racket Sport Research, Institute of Exercise Training and Sport Informatics, German Sport University Cologne, Cologne, Germany; ^2^Department of Coaching, Health and Physical Education, School of Sport and Recreation, Auckland University of Technology, Auckland, New Zealand

**Keywords:** specialist coaching, skill acquisition training, motor learning, constraints-led approach, movement adaptability, representative training, soccer goalkeeping

## Abstract

Across sports and movement science, training periodization has been recognized as key for athlete development and performance. While periodization with regard to physiology has a proven history, the structuring and periodization of motor learning and skill development is seemingly less researched and practiced. Despite the existence of numerous theoretical accounts underpinning skill acquisition training and more recently emerging periodization models, a cohesive framework to practically support coaches in the context of “specialist coaching” appears to be needed. The use of “specialist coaches” for individualized, one-on-one or small group trainings displays a growing trend in team ball sports. Despite limiting the replication of game-representative environments (i.e., by constraining the number of involved athletes in training), “specialist coaches” in performance sport constantly aim to achieve marginal gains and refinements in athlete development. In order to support these “specialist coaches” and fill a research gap on skill training periodization, the current paper seeks to review and transfer contemporary skill acquisition training theory (driven by the constraints-led approach) into a practically-applicable “Periodization of Skill Training” framework (“PoST” framework). This framework provides valuable conceptual and practical support for “specialist coaches” in performance sport; which will in turn, enhance, and refine adaptive movement variability for sport skills and manipulate skill training environments (i.e., over the course of macro- and micro-cycles, and for the planning of single training sessions). Practical examples from soccer goalkeeping (i.e., a “specialist coaching” context, often constrained to a small number of players in the training environment) will underline the proposed framework.

## Introduction

Across team sports, it is commonly recognized that organized training activities (led by coaches) enhance athlete development and better prepare performers for the dynamic demands of competition environments (Hodges and Franks, 2002; Côté et al., 2007). These training activities, usually in the form of “team training” (e.g., 22 players in a soccer training session), are acknowledged to be most effective for skill acquisition and refinement when replicating the complex interacting constraints that are representative of performance environments (Davids et al., 2008; Renshaw et al., 2019a). Yet, in order to achieve marginal gains when developing athletes, there is a growing trend in performance sport for the use of “specialist coaches” for individualized one-on-one or small group trainings (Smith, 2018). The involvement of “specialist coaches” has been common practice in sports like American football, basketball and rugby for years; however, specialized coaching positions in soccer, such as a “striker coach,” “throw-in coach,” “rehab coach,” or “individual development coach,” have only more recently emerged (e.g., BBC, 2018; Smith, 2018; Austin, 2019). Given the prominence of the “specialist coaches” trend, supporting them with up-to-date scientific principles on skill training planning appears to be important. The involvement of a limited number of athletes often proves to be a constraint in “specialist coaching” training environments, and so the challenge of full representation of the performance demands may be denied. Thus, informational constraints in these training environments may not invite exploration of action opportunities. Following this limitation, and driven by anecdotal evidence and abiding scientific concerns about the coaches' choice of training activities, we aim to circumvent the risk of “specialist coaches” moving toward “traditional” coaching methods. These “traditional” coaching approaches are often based on intuition (Williams and Ford, 2009), “folk pedagogies” (Harvey et al., 2013), and/or have historical precedence (Williams and Hodges, 2005). In detail, several features may be displayed by “traditional” coaching: (1) limited performance uncertainty and variability of actions (Passos et al., 2008; Krause et al., 2019); (2) decontextualized movement coordination from the performance environment (Stolz and Pill, 2014); and (3) monotonous and repetitive technical drills in training (Renshaw et al., 2009; Krause et al., 2018).

Realizing concerns about the application of contemporary coaching approaches, we aim to meet recent calls from multiple directions for advancement in coaching practice. On the one hand, there is a need for “more engagement of researchers in supporting the application of theory into practice” (Renshaw et al., 2016, p. 475); and on the other hand, there has been recent demand for coaching practitioners to adopt research-supported coaching methodologies in order to systematically plan (and periodize) skill training (Correia et al., 2019; Renshaw and Chow, 2019). With the calls for more support from researchers in mind, there have been some attempts to look at the periodization of skill training; in particular, the Skill Acquisition Periodisation (SAP) framework by Farrow and Robertson (2017). The “SAP” framework was developed to measure and monitor longitudinal skill training in a high performance environment. These authors did a remarkable job at re-conceptualizing and relating physiology periodization principles to their “SAP” framework in order to help coaches understand the concepts more easily; for example, using existing and already-understood training physiology terms like specificity, progression, and overload. In addition, the “SAP” framework does an excellent job of separating aspects of the training environment (e.g., specificity or progression) and proposes measurements for these aspects. While many of these measurements are well-described in the 2017 paper, both sub-elite and elite performance sports coaches may struggle accessing relevant equipment, specialists, or having the time for the proposed measurements.

Farrow and Robertson's outlined strategies for longitudinal skill training of athletes in a high performance environment look to be particularly useful for team coaches that run full year or multi year programmes in preparation for international events. However, there are many situations (such as those experienced by a “specialist coach”) where a practitioner will only work with an athlete for a single session or a short period of time; in such situations, longitudinal periodization is ultimately aimed for, but may not be possible given the constraints of the training environment (e.g., the time with the athlete). The “Periodization of Skill Training” framework (referred to as “PoST” framework) presented in this paper (see later) aims to integrate the skillful monitoring and measuring principles of the “SAP” model. In order to advance the field of skill acquisition training for those not in charge of whole teams, the proposed framework presents a model of skill development and refinement. This framework is especially targeted at “specialist coaches” working at the performance level with non-representative settings and very small athlete numbers.

Next to the “SAP” framework, a prominent framework for skill learning (and training periodization) is displayed by game-based approaches (GBAs); these, in large, describe “the use of modified and/or conditioned games” to holistically evolve game-intelligent performers (Kinnerk et al., 2018, p. 6). To date, numerous GBAs, such as the Teaching Games for Understanding (TGfU) Model or the Game Sense Model (GSM), have been proposed and applied to team sports contexts (see Kinnerk et al., 2018, and Stolz and Pill, 2014, for detailed reviews of various GBAs). In detail, GBAs are theoretically grounded on constructionist learning theory and discovery learning (Bruner, 1979), and originate from an educative-pedagogical perspective (Harvey et al., 2018). While highlighting a learner-centered approach (as opposed to a learner environment-centered approach in the constraints-led approach; Renshaw et al., 2016), GBAs consider a variety of playing forms (Pill, 2012, 2016); these forms include: “small-sided games” (i.e., “match-play with reduced number of players and two goals”; Ford et al., 2010, p. 487); and “conditioned games” (i.e., “small-sided games, but with variations to rules, goals or areas of play”; p. 487). Despite some theoretical distinctions to the constraints-led approach (see Renshaw et al., 2016; Harvey et al., 2018), we do applaud the contribution of GBAs for the modification of skill training for entire teams and large groups of athletes. However, regarding the context of “specialist coaching” with single athletes, applicability of the aforementioned playing forms and games is limited to team-based training approaches (see later).

Considering previous enriching conceptual work and its potential limitations, it remains important to further progress and translate academic knowledge in order to support coaches in their systematic planning of “real world” training sessions. Thus, we aim to provide theory-driven challenges and practically-applicable tools for coaches to systematically plan and adjust task constraints in individualized training with skilled athletes (see later); i.e., over multiple training months, weeks and for units within single training sessions. While widely-advocated theoretical groundwork is considered throughout this paper, we apply it to a newly emerging training context. In detail, we consider “specialist coaches” working on position-specific details with performance athletes (individually or in small groups). By proposing the “PoST” framework (grounded on ideas from the “SAP” model), we encourage practitioners to actively assess (1) individual athletes' skill levels and training stages; (2) the training environment based on the number of athletes involved; and (3) perceptual-cognitive and motor demands that athletes face in dynamic performance environments (i.e., the game). Finally, while the “PoST” framework and its (sub-) stages introduce the concept of “information complexity,” it further supports a coaching practitioner focus by proposing approaches toward replicating (parts) of game-representative demands within session designs. These approaches are explored based on two critical conceptual questions: how can coaches adequately facilitate and periodize skill training environments (1) over the course of a macro-cycle and micro-cycle (i.e., the periodization of “specialist” training over the course of multiple months and sessions during 1 week of a season)? and (2) throughout a single training session (i.e., the structure of a single “specialist” training session with ~1–4 athletes at the performance level)?

In an attempt to present contemporary theoretical insights, and apply these to the “PoST” framework for “specialist coaching,” this paper is structured in three distinct parts: Part A, skill training theory and research, which provides a theoretical foundation for part B, “Periodization of Skill Training” framework (“PoST”), which intends to introduce a novel and practically-applicable approach to skill training periodization in the context of “specialist coaching”; and Part C, application of the “PoST” framework to training planning. This latter section (C) introduces practically applicable tools for sports coaches to use when periodizing and planning skill training with individual athletes or smaller groups of athletes (i.e., the current paper focuses on one to four athletes). Skill training, for this paper, is where the athlete is able to “seek, explore, discover, assemble, and stabilize the coordination of movement patterns” (Davids et al., 2008, p. 83), and training periodization is defined as systematic “short- and long-term planning to prescribe specific workloads and tasks” for athletes (also interchangeably termed as “learners,” “performers,” and “players” throughout this paper) (Farrow and Robertson, 2017, p. 1044).

Throughout the paper, the “specialist coaching” context of soccer goalkeeper (GK) training will be used as a vehicle to elaborate on the presented “PoST” framework. This particular context is chosen for various reasons; these include: (1) GK coaching is a very specialist coaching area, which sees GK coaches often working with player groups involving between one and four athletes; and (2) there appears to be continued prominence of traditional coaching approaches in GK training, which arguably limit representativeness (see Williams and Hodges, 2005). For example, in a recent study by Otte et al. (2019) using qualitative interviews, professional soccer GK coaches indicated each training session to typically follow a linear structure, where soccer-representative “complex activities” only account for around 30% of the total session time (as compared to 50–70% focusing on isolated technical work). This finding would further support the claim by Renshaw et al. (2010) that coaches need not only to individualize and systematically plan their skill training programmes for athletes, but also to maintain a learning environment that replicates dynamic performance situations.

## PART A. Skill Training Theory and Research

In order to attempt to connect and apply academic knowledge to the “real coaching world,” part one will briefly review the constraints-led approach (CLA) as a perspective on skill (acquisition) training; and part two introduces the role of the coach in managing the training environment by specifying three theory-driven challenges for practitioners.

### CLA as a Theoretical Account on Skill Training

From a theoretical perspective, the CLA (underpinned and framed by dynamical systems, ecological psychology and non-linear pedagogy) will be adopted in order to review the acquisition of skills and its training periodization (see Renshaw and Chow, 2019; Renshaw et al., 2019b, for recent overviews of the CLA in skill acquisition training). In brief, the CLA, as an ecological model, advocates the underlying principle that open systems (e.g., athletes within invasion games or team ball games) are of non-linear nature and involve a mutual relationship with the performance environment (Pinder et al., 2011; Renshaw et al., 2016). During soccer games, for example, the GK's interception tasks (e.g., catching a shot on target) are dependent on numerous complex constraint interactions. These interpersonal interactions emerge from other players' movements and actions, as well as from further task (e.g., game score), environmental (e.g., changing weather or light conditions), and individual constraints (e.g., motor system fatigue, motivation, or emotions) (Davids, 2012, 2015; Correia et al., 2019; Renshaw et al., 2019b). This idea may lead to the notion that the game environment in open play situations at different points in time provides constantly changing constraints on the performer and thus, situations can never be identical (Renshaw and Chow, 2019). Consequently, GKs' self-organized and functional movement solutions will always have to be adapted slightly and contain internally-induced movement variability (see Kelso, 1995, for a theoretical overview); to provide an example: a GK catching a central shot on a rainy day (as compared to a sunny day) may demand different movement adaptations (e.g., arm and hand acceleration) in order to successfully achieve the aim of saving two comparable shots from similar distances.

Due to the ever-changing constraints on the performer, the CLA stresses two prevailing aspects (amongst others): (1) the athlete's ability to adapt and perform self-organized functional movement solutions in response to the emerging combination of task, environment, and individual constraints; and (2) the athletes' constant coupling of information and perception with movements and actions (Newell, 1986; Renshaw et al., 2010). The former aspect can be underpinned by Bernstein (1967) “degrees of freedom problem,” which demands performers to temporarily control relevant motor system degrees of freedom, so as to develop functional coordinative structures and “to ensure that movements are adapted to changing circumstances of performance” (Davids et al., 2008, p. 43). The latter aspect links to Gibson (1979; 2019) theory of affordances (i.e., opportunities for action and behavior, which act as information for motor executions and decision making in sport; Renshaw et al., 2010; Van Der Kamp et al., 2018). Emerging from “continuous interactions of an athlete with key features of a performance environment” (Davids, 2015, p. 53), these affordances are both body-scaled (i.e., depending on individual performer's action capabilities, such as technical abilities or fitness level) and action-scaled (i.e., depending on emergent environmental properties, such as teammates' movements; see Fajen et al., 2008, for a detailed review).

To relate the theoretical perspective of the CLA (in connection with its theoretical underpinnings) back to the subject of skill training and its periodization, it appears to be essential for coaches to gain an understanding of how they can organize and manipulate task constraints, so as to allow athletes to explore and discover relevant information within the training environment (Renshaw et al., 2016). Thus, the CLA provides a theoretical perspective, which can allow coaches to meet individual athlete's needs by manipulating the learning environment.

### The Role of the Coach in Managing the Training Environment

The coaches' ability to provide athletes with appropriate affordances (primarily, via the use of task constraints) within game-representative training environments is incumbent for skill learning. In order to facilitate an appropriate training environment, there appear to be three key challenges for “specialist coaches” to manage and make decisions on; these include the key concepts of (1) the *representativeness of training*; (2) *stability and instability in training*; and (3) the *level of information complexity* (i.e., as managed by task constraint manipulations and the practice schedule of movement tasks). In detail, the former two challenges support training designs in terms of their “*replication value”* of actual performance demands and preparation for movement adaptability in dynamic team sports. The latter challenge helps practitioners to manage the “*level of appropriateness”* of the training environment, in relation to the performer's perceived task complexity and skill level.

#### Challenge 1: Introducing Representativeness and Task Specificity in Training

The merit of *representative learning designs* (i.e., measured by the degree to which skills acquired in practice transfer to the competitive environment; Renshaw and Chow, 2019) has been supported by an extensive body of research (see Pinder et al., 2011; Krause et al., 2018, 2019). In detail, representative training designs display three dominant notions: (1) providing learners with relevant affordances and perception-action couplings, so that they become perceptually attuned to critical information sources (e.g., Seifert et al., 2017; Orth et al., 2019); (2) facilitating training that holistically integrates motor processes (e.g., specific techniques) with perceptual-cognitive components (e.g., decision-making and tactical awareness; Ford et al., 2010; Broadbent et al., 2015; Farrow and Robertson, 2017); and (3) developing training designs that recreate action fidelity (i.e., the degree of correspondence between emerging behavior in a training task compared to competitive performance; Travassos et al., 2012) and functionality (i.e., the achievement of goals in the performance environment that are based on actions and constraints that athletes have been exposed to in the learning context; Pinder et al., 2011). Scientific evidence has found that athletes of dissimilar performance and development stages perceive different affordances. More advanced performers (across different sport contexts) appear to be perceptually better attuned and so tend to perceive more relevant information from the environment (e.g., Button et al., 2003; Travassos et al., 2012). Consequently, the idea of replicating dynamic competition demands in training, considering various levels of game-representativeness (see later), lies at the heart of the proposed “PoST” framework.

#### Challenge 2: Finding a Balance Between Stability and Instability

Movement adaptability explains the ability to produce and coordinate an appropriate ratio of stable and unstable movement behavior when required (see Renshaw et al., 2009; Seifert et al., 2013, for in-depth discussions of the dynamical systems theory applied to movement behavior in sports). While *movement stability* states the maintenance of a system's coordinative structure under perturbation, *instability* represents exploitation of fluctuations, so as to develop a functional response to perturbations caused by uncertainties in the dynamic environment (Conrad, 1983; Seifert et al., 2013). Along this continuum of maintaining the stability of actions (that provides a structure to performance and may enhance performer's motivation and confidence) and creating instability of actions (that leads to adaptive functional movement variability), “specialist coaches” have the opportunity to influence athlete's self-organization processes (Handford, 2006; Passos et al., 2008; Renshaw et al., 2009). Particularly, it is paramount that coaches systematically manipulate task constraints (e.g., goal or ball sizes) in training to intentionally create situations that lead to a change in an athlete's coordination (Renshaw and Chow, 2019). These directed and promoted changes, in particular, can be framed by the concept of “degeneracy,” which describes achievement of the same output while (structurally) varying motor behavior (Renshaw et al., 2016); hereof, athletes learn to exploit various performance solutions and the stability of actions under dynamic and perturbing task constraints (Seifert et al., 2013). In simple terms, coach-induced coordination changes in training aim at enhancing an athlete's ability to achieve the same task goal in different ways and support “the search for, exploration of, and exploitation the use of the same solution to respond to different problems” (Correia et al., 2019, p. 124).

#### Challenge 3: Managing the Level of Information Complexity in Training

A process or task goal (e.g., the coordination of movement within a dynamic ball game) may be subjectively perceived as complex by an individual if “the amount of information required to control the process is large” (Backlund, 2002, p. 34). Adopting this description of complexity, its qualitative nature based on individually perceived dynamic system interactions must be highlighted (Davids, 2012). In particular, Yates (1978, 1987) states several attributes found in complex processes; these include: non-linearity, high numbers of degrees of freedom, and active interactions among parts and actors within the environment. All of these attributes underline the view that athletes are non-linear movement systems with inherent self-organization tendencies and mutual performance-environment interactions (Phillips et al., 2010; Davids, 2012, 2015; Seifert et al., 2013, 2017). Therefore, applying the concept of “information complexity” (interchangeable termed as “task complexity”) to skill training in a “specialist coaching” setting appears to be appropriate. In particular, “specialist coaches” ought to manage informational complexity that challenges athletes' perception-action couplings while performing specific training tasks. By highlighting, dissimulating or expanding on perceptual-cognitive variables (e.g., the movement speed of a dribbling attacker), coaches may constrain the affordances available for individual athletes' exploration within the given training environment (Davids, 2015).

Notably, a particular challenge for coaches is stressed by the notion of similar training tasks being more or less feasible for different performers (i.e., according to their possibilities to successfully explore existing affordances). This idea aligns with Stoffregen (2003) definition of affordances as emergent properties of animal-environment systems and thus, states the subjectively perceived level of information complexity to create different challenges for each individual performer. In order to “provide an appropriate level of challenge” for individualized skill learning (Farrow et al., 2008, p. 497), coaches are therefore required to: (i) modify task constraints and equipment; and (ii) manage practice task schedules.

##### Task and equipment modification to manage information complexity

Alongside manipulating commonly advocated task constraints, such as instruction, rule changes, or playing area and surface adjustments (Correia et al., 2019), benefits of modifying equipment for the management of informational complexity are proposed. In particular, the removal or addition of perceptual information is considered to support or challenge athletes' exploration for functional perception-action couplings and movement solutions (Davids et al., 2008; Renshaw and Chow, 2019). These task constraint manipulations (i.e., to lower or increase perceived task complexity) may be beneficial for several reasons. Firstly, by modifying equipment, perceived task complexity may increase (e.g., differently weighted and shaped balls may cause less predictable flight and bounce patterns and increase task complexity for catching). Thus, modifications may support learners in organizing movement system degrees of freedom without disrupting subordinate levels of the central nervous system (Hodges and Franks, 2002; Davids et al., 2008). Specifically, added equipment may distract learners from consciously attending to and reinvesting in explicit details of movement mechanics (since a detailed elaboration of the “reinvestment theory” and the vast body of research in sport supporting the theory would go beyond this paper's scope, see Masters and Maxwell, 2008, for an overview). Consequently, implicit coordination of movement solutions and implicit learning processes, which have been shown to enhance robustness of skills under performance pressure, may be enhanced (Jackson and Farrow, 2005; Panchuk et al., 2014). Secondly, task manipulations based on equipment variation may guide athletes' visual search processes toward most critical information sources within the environment (e.g., an attacker wearing colored markers may guide GKs' gaze behavior toward critical postural and kinematic information sources, such as hip orientation and position; e.g., Ryu et al., 2013). Thirdly, added equipment may direct athletes toward alternative sensory and perceptual information (e.g., when impairing the visual array through specialized glasses, athletes' actions may need to be more strongly supported by acoustic arrays within the environment; Davids et al., 2008). Notably, despite proposed benefits of manipulating information complexity for the athlete(s) via equipment into skill training, “specialist coaches” need to be cautious about how and when to use training aids to support learning goals effectively.

##### The practice schedule of movement tasks to manage information complexity

The practice schedule describes the number of movement tasks and the order in which they are required to be performed by the athlete (Wulf and Shea, 2002). Due to this paper's primary focus on “specialist coaching” at the performance level (i.e., focused on skill refinement of complex skills executed by athletes in team sports), two distinct practice schedule arrangements are considered: “within-skill variability” (i.e., “discernible variation in the execution of the same skill”); and “between-skill variability” (i.e., “switching of skills during practice”; see Buszard et al., 2017, p. 2). While the former arrangement is likely to present an intra-task interference condition, the latter arrangement displays inter-task interference conditions. Notably an increase in information complexity may result from adjustments of both practice schedule arrangements.

From a CLA perspective, increasing both intra-task and inter-task interference (and thus, information complexity) may lead athletes to explore the training environment for more relevant information, thereby forming stronger perception-action couplings (Davids et al., 2008; Renshaw et al., 2019b). Research often advocates the benefits of increasing interference in order to encourage movement adaptations and enhance skill learning (Davids et al., 2008; Orth et al., 2019); particularly, this notion has been claimed to be advantageous for “skilled performers refining complex motor skills in applied environments” (Buszard et al., 2017, p. 11). While practice schedule manipulations may result in the demonstration of more unstable movement coordination in the short term, in the long-run, the induction of perturbations to performers' perceptual-motor landscapes supposedly leads to more robust coordination structures (i.e., often termed as “attractors”); these structures can be applied to dynamic environmental changes by performers in team sports (Davids et al., 2008; Renshaw et al., 2010). Despite this perspective on advocating increased interference and variability in training, coaches may use practice schedules of movement tasks to regulate information complexity that performers are confronted with. The need for “specialist coaches” to consider the practice schedule is also strongly highlighted in the “SAP” framework (Farrow and Robertson, 2017) in terms of specificity of training and tedium challenges.

## PART B. “PERIODIZATION of Skill Training” Framework

**Figure 1:**
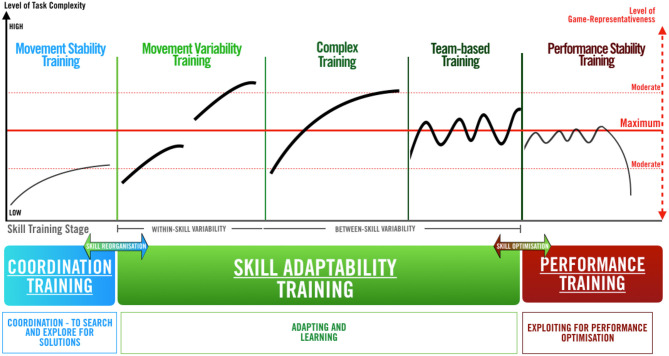
The “Periodization of Skill Training” framework (“PoST” framework).

Part B, “Periodization of Skill Training” framework (“PoST”) (see Figure 1), presents and elaborates on the framework's underlying structure, which is informed by above-mentioned theoretical insight, and has a particular focus on the second motor learning stage of “Skill Adaptability Training” (see below). Practical examples from soccer goalkeeping (i.e., a “specialist coaching” context, often constrained to a small number of players in the training environment) will be used to illustrate the training (sub-) stages.

### Structure of the “PoST” Framework

Decades of research on motor skill learning have proposed numerous descriptive models of the process of skill acquisition. One common and ecological psychology-advocated model is Newell (1985) “model of motor learning,” which is the foundation of the “PoST” framework. Newell's original model proposes three stages of motor learning (i.e., “skill coordination stage”; “skill control stage”; and “skill optimization stage”); these are based on Bernstein (1967) work on human movement systems (see Davids et al., 2008, for a detailed discussion of the model to dynamics of skill acquisition). Derived from the CLA perspective and in relation to Newell (1985) model, the “PoST” framework displays three main skill training stages (i.e. “Coordination Training,” “Skill Adaptability Training,” and “Performance Training”). For this paper, particularly the second stage (i.e., the “control stage” of coordinative structures, or termed as “Skill Adaptability Training”) will be highlighted in terms of skill training and learning.

Overall, when planning or periodizing skill training, “specialist coaches” are encouraged to focus on two main areas. Firstly, coaches ought to focus on a relevant skill training stage for the individual athlete (i.e., the x-axes of the graph). Secondly, coaches need to carefully manage the level of task representativeness (see challenge 1, and the right-hand y-axis) and perceived task complexity (see challenge 3, and left-hand y-axis). In relation to the demands of the actual performance environment and individual athlete's capabilities and skill level, the framework provides a bi-dimensional representation of these factors. While the red dotted y-axis (i.e., on the right in Figure 1) displays a bi-directional measurement of game-representativeness, the black y-axis (i.e., on the left in Figure 1) presents the athletes' perceived level of task complexity. In detail, game-representativeness is stated to progress and regress, in order to highlight the training design's “*replication value”* of the actual performance environment (i.e., displayed by the horizontal, red solid line showing a maximum level of game-representativeness). Task complexity, in contrast to the primary y-axis, progressively increases in worth from “low” to “high” (i.e., the black curves on the graph); this progressive development aims to account for the subjective nature of individual's perception of information complexity (e.g., while a training task remains at a moderate game-representativeness level, this task may be perceived as low in information complexity by GK A and high in complexity by GK B).

Notably, for coaches to further manage the level of perceived task complexity and facilitate a learning environment that is appropriate for the individual athletes, movement back and forth between the skill training stages (i.e., on the x-axis) should be considered. In particular, practitioners may need to facilitate “skill reorganization processes” (i.e., (re)freezing motor system degrees of freedom to induce movement stability) and “skill optimization processes” (i.e., the functional grouping of an increased amount of motor system degrees of freedom) (Bernstein, 1967; Davids et al., 2008). The horizontal movements between various skill training (sub-)stages for single learners are supported by basic tenets of non-linear pedagogy; these, in particular, highlight: (1) motor learning as a non-linear process (i.e., athletes show different rates of skill learning and different time scales for progression; e.g., Phillips et al., 2010; Renshaw and Chow, 2019); (2) the need for individualized and varied pathways for learning (e.g., Chow, 2013); and (3) the absence of a single best way of learning and teaching (i.e., the same training approach may affect individual performer's learning differently; e.g., Correia et al., 2019).

#### “Coordination Training”

The first (left) part of the skill training framework (i.e., “Coordination Training”) is focused on searching for and exploring coordination movements within the emerging training environment. In order to acquire basic movement patterns and stable coordination structures, performers in this stage should experience rather low levels of environmental variability and task complexity (i.e., the gray line in this sub-stage remains at a moderate or lower level of game-representativeness; Renshaw and Chow, 2019). Particularly, the method of task simplification may be useful for coaches in this skill training stage. This approach of using “scaled-down versions of tasks [that] are created in practice and performed by learners” (Chow et al., 2007, p. 270) aims at maintaining relevant information-movement couplings and constraint interactions, while blocking degrees of freedom in order to meet the athlete's skill level (Davids et al., 2008; Correia et al., 2019).

With regards to the soccer GK context, this phase would predominantly focus on the acquisition of fundamental movements (e.g., catching and diving), while retaining intact player-environment interactions; for example, a coach may dribble and shoot slowly on a GK—this would allow the GK to get set, perceive the moment of ball-foot contact (for the shot) and time movement coordination toward catching the shot. Coaches would allow learners to repeatedly freeze motor system degrees of freedom under constant environmental conditions in order to manage the task demands and achieve task outcomes (Seifert et al., 2013; Buszard et al., 2017). Immediately upon developing (some) movement stability, coaches are encouraged to change the training structure and organization (Wulf and Shea, 2002; Farrow and Robertson, 2017). In other words, movement consistency under controlled environmental conditions over a set period of time (e.g., several training sessions) would be regarded as a trigger for coaches and athletes to (more permanently) progress to the second skill training stage of “Skill Adaptability Training.”

#### “Skill Adaptability Training”

With the aim of enhancing the adaptability, functionality, and robustness of motor skills under perturbation of dynamic environments, the second development phase, “Skill Adaptability Training,” focuses on skill learning. Here, performers are challenged to self-organize and adapt coordinative structures with complex and representative constraints of the dynamic performance environment (Davids et al., 2006; Araújo and Davids, 2011; Renshaw and Chow, 2019). The idea that coordinative structures become more open to constantly changing information sources and environmental perturbations drives this non-linear training stage (Davids et al., 2008).

In order for coaches to encourage movement variability and enhance skill learning, the “PoST” framework proposes three sub-stages (see Figure 1); these stages are termed as: (1) “Movement Variability Training” (i.e., the first green section from the left in the framework); (2) “Complex Training” (i.e., the second green section from the left); and (3) “Team-based Training” (i.e., the third green section from the left).

##### “Movement variability training” stage

The first sub-stage of “Skill Adaptability Training” focuses on individualized training contexts consisting primarily of one (or two) athletes and a “specialist coach” (i.e., notably, the training environment is constrained in terms of game-representativeness, due to the low number of participants). Performers, here, focus on movement variability, which aims to enhance the ability to adapt movement parameters in response to changing constraints in the environment. This sub-stage is driven by the aim of challenging performers to more actively search for relevant information sources and adapt micro-component features of movement solutions (i.e., “within-skill variability”). Predominantly, this would be within a stable affordance landscape and under varying levels of task complexity. In other words, while the practice schedule of movements is largely known to the performer, motor executions need to be flexibly adapted under changing constraints.

**Figure 2:**
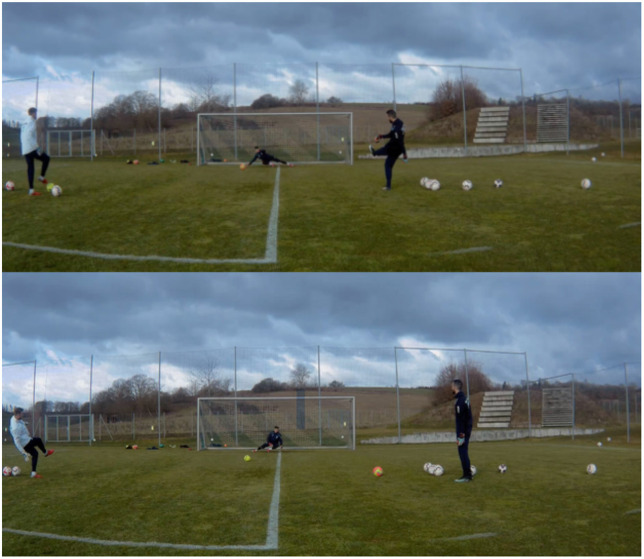
“Movement Variability Training” example: the GK is asked to repeatedly dive sideways and deal with low shots. While the coach(es) constantly manipulate(s) object parameters and the trajectory by using different sized balls and various shooting and throwing techniques, further perceptual-cognitive interference may be added by wearing special glasses to limit the GK's vision. Notably, the line in the middle of the goal (as a task constraint) aims at supporting the GK by providing additional spatial orientation.

Methodical approaches within this stage of “Movement Variability Training” could include, for example, the differential learning approach or the addition of changing parameters and modified equipment to the movement task at hand. The former approach demands learners to perform each movement repetition with slight “fluctuations” (i.e., variations). These fluctuations lead individual performers to search for a movement optimum from the entire range of possible intra-movement variations (e.g., Schöllhorn et al., 2009; Farrow and Robertson, 2017). The latter methodical approach of inducing perceptual-cognitive interference, on top of confronting learners with movement tasks, aims to increase/decrease task complexity and challenge exploration of critical informational sources emerging from performer-environment interactions. In particular, these exploratory processes may be initiated in different ways; for instance, by adding task constraints (e.g., by increasing time or opponent pressure and changing object parameters). Furthermore, modifying equipment in order to support or limit sensory perception could add benefits (see challenge 3). For example, Figure 2 (below) displays a soccer GK training session with an individual GK that focuses on the single movement task of “diving sideways.” While the GK is confronted with this movement task, the coach could manipulate invariant and variant information variables, such as object parameters and the trajectory (e.g., by adjusting the ball speed, ball size, or shooting distance) and add further informational complexity through inducing perceptual-cognitive interference (e.g., special glasses limiting peripheral vision). In turn, this would challenge the GK to vary spatiotemporal kinematic movement parameters, such as arm acceleration and timing toward the ball, while also having to deal with supplemental visual limitation.

Overall, this first skill learning sub-stage focuses on single movement tasks under perceptual-cognitive interference, and thus, the level of task complexity may either fall short of or exceed the demands of competition (i.e., the sub-stage displays two black curves below and above the red solid line, respectively). Despite acknowledging that aforementioned perceptual-cognitive interference may make the training environment less game-representative in terms of superficial similarities (i.e., the black lines do not intersect the red solid line), we propose that these modifications may have beneficial effects for skill refinement (see challenge 3).

##### “Complex training” stage

Transitioning from the first sub-stage (focusing on single movement tasks in training environments including one or two athletes), the “Complex Training” stage aims at confronting performers with multiple movements via further increase of information complexity (e.g., small training groups consisting of up to four athletes in soccer goalkeeping). At first, these movement tasks may share common features and structural task similarities (Braun et al., 2009; Braun, 2013); examples, hereof, could contain shared movement sequences and interception tasks (e.g., the GK's arm and hand movement and acceleration toward catching a high cross or a central shot above the athlete's head) or shared perceptual tracking requirements used to develop functional perception-action couplings (Hebert et al., 1996). By limiting movement tasks to those with structural commonalities, athletes could be encouraged to explore a smaller perceptual-motor landscape, while continuously trying to adapt movement solutions and achieve the task outcome. Considering these rather stable training conditions, task complexity, at first, may be perceived as low to moderate by athletes (i.e., the low part of the black curve in the second green sub-stage from the left). The level of game-representativeness may not quite reach the red solid line (i.e., maximum game-representativeness).

**Figure 3:**
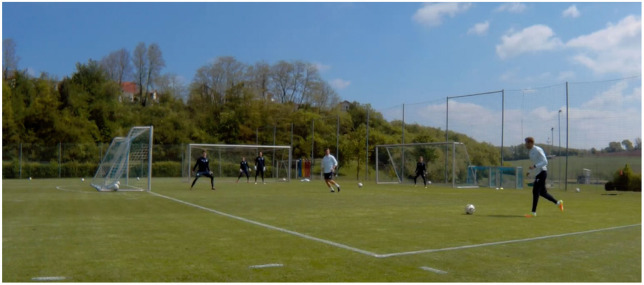
“Complex Training” example: a GK-specific shot-stopping exercise that requires three GKs to defend the three goals. Further GKs act as attackers in the center of the field, in order to increase task complexity and representativeness.

**Figure 4:**
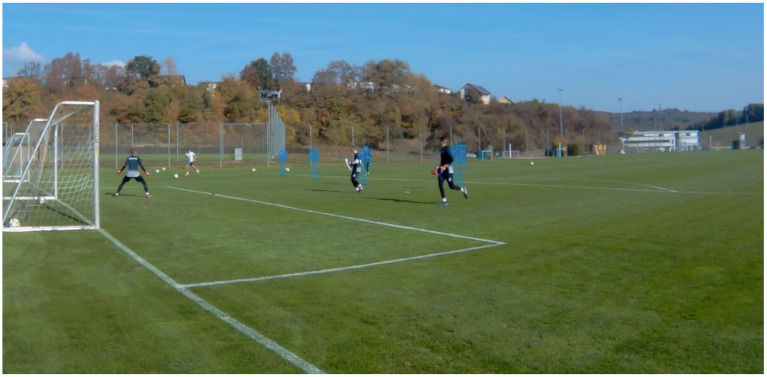
“Complex Training” example: complex training exercise in a GK-specific training session with four GKs. A break-through situation inside the box results in a “cut-back pass” into the middle or a direct shot on goal. The GK is required to perceive relevant information variables (e.g., the attacker on the ball) and exploit the given affordances, so as to achieve the task of defending the goal.

Moving up to the black curve in this “Complex Training” sub-stage, practice task schedules may highlight “between-skill variability” of unrelated movement skills. Driven by the modification of various task constraints, athletes' perceived task complexity may increase significantly. For example, training tasks in which the coach or further attackers could shoot on goal from various angles and distances would require the GK to perceive relevant kinematic information from various sources (e.g., multiple possible shooters). Additionally, GKs here would be challenged to explore an increased number and complexity of affordances within the dynamic training environment and thus, have to effectively couple information with movements (see Figures 3, 4 for examples). Due to constraint manipulations, such as increased intensity and loading (e.g., leading to fatigue), stress (e.g., opponent pressure), and the number of repetitions experienced (within a relatively short time), the later phase of this sub-stage may work above the red solid line (i.e., the upper part of the black curve in the second green sub-stage). While containing representative elements (e.g., the perception of kinematic information from a shooting attacker), an exact replication of game demands may only be achieved to a certain extent (e.g., in the attacking situation, the GK may only be required to intercept shots or crosses from a predictable range of distances and angles; Figure 4). Despite the limited number of game-representative affordances, these training forms are likely to be perceived as highly complex by athletes.

##### “Team-based training” stage

Under the umbrella term of “Team-based Training,” this third sub-stage aims to closely replicate game-representative performer-environment interactions according to principles from CLA and non-linear pedagogy (Renshaw and Chow, 2019). In contrast to the above-mentioned context of “specialist” individual or small group coaching, this training stage refers to training with an entire team or larger groups of athletes (e.g., four or more athletes in soccer training). Consequently, with an increased number of athletes involved in training, facilitating game-representative affordances arguably becomes significantly more accessible for coaches. In particular, by re-introducing aforementioned “playing forms” (e.g., small-sided games; see introduction), athletes are likely to be confronted with complexity of information that matches the complexity perceived in games (i.e., working closely around the red solid line); thus, coaches may attune athletes to relevant action opportunities. In order to further elaborate on this idea, we adopt previously-established categorizations of “playing forms” in team sports; these, for the purpose of this paper, include (1) “phase-of-play situations”; and (2) “conditioned games,” “small-sided games,” and “larger games” (see Partington et al., 2014; O'Connor et al., 2017, for detailed discussions).

**Figure 5:**
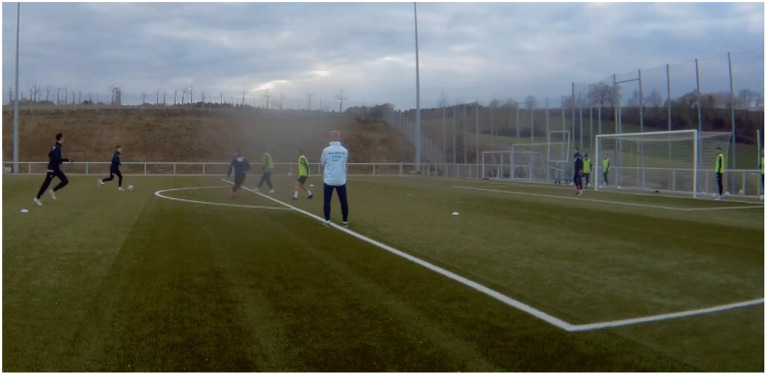
“Team-based Training” example: a “phase-of-play situation” presenting a 3v2 on one goal. The GK is required to respond to the emerging interactions between the attacking and defending team in order to coordinate functional movement solutions.

Firstly, “phase-of-play situations” describe “uni-directional match-play toward one goal [or target]” (Ford et al., 2010, p. 487). Training of game-representative situations, in particular, may include repeated simulations of attackers penetrating an opponent's defense in order to create goal scoring opportunities (O'Connor et al., 2017). Thus, constraints that are frequently found within performance environments are repeatedly explored; for example, Figure 5 presents a 3v2 attacking situation at the edge of the goal box in soccer that confronts the GK with emerging attacker-defender interactions.

**Figure 6:**
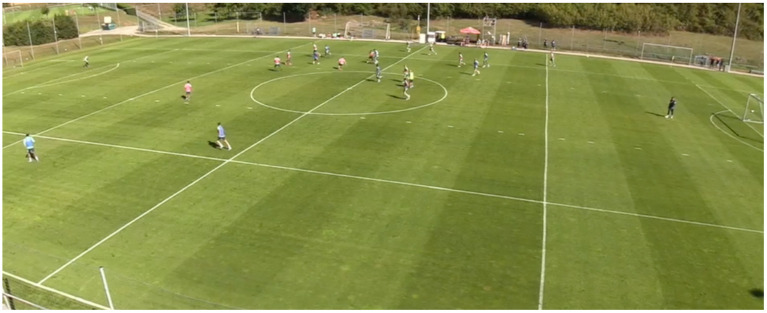
“Team-based Training” example: a ‘larger game' presenting an 11-vs.-11 soccer training game played on a shortened soccer pitch. In addition to the task manipulation of the field size, further line markings across the field aim to constrain the playing surface and support players' tactical positioning during the game.

Secondly, a three-fold differentiation for training games is proposed; these include: (1) “conditioned games”; (2) “small-sided games”; and (3) “larger games” (i.e., games “during training where players work in teams of 5 or more”; O'Connor et al., 2017, p. 650). These playing forms aim to facilitate an environment in which performers or teams compete against each other in (free) play, so as to develop task-specific and adaptable coordination patterns (Broderick and Newell, 1999; Rink, 2001; Davids et al., 2008, 2013). While “conditioned” and “small-sided games” strongly promote exploration of interpersonal interactions, they often constrain players in regard to space, time and player numbers included (Davids et al., 2013; O'Connor et al., 2017). Consequently, the additional use of “larger games” for replication of game actions appears to be another cornerstone of this skill training sub-stage. Figure 6, for example, displays a “larger game” consisting of an 11-vs.-11 soccer training game played on a marginally shortened soccer pitch. This training organization, although harder to destabilize (as compared to small-sided games; Davids et al., 2013), arguably displays one of the highest levels of representativeness (in regard to the demands that the GK will face in competitive soccer games).

In sum, while training games themselves drive learning, it is the coaches that further facilitate the training environment by manipulating task constraints (e.g., field size, rules, equipment) and by driving feedback processes through instructional strategies of questioning and guided discovery (Chow, 2013; Renshaw et al., 2016). As indicated by the wavy black line (i.e., the dark-green sub-stage second from the right in the framework), it is this particular sub-stage of “Team-based Training” that works to closely replicate actual game demands (i.e., the wavy black line intersects the red solid line). Thus, this skill training sub-stage presents one (if not, the most) critical component of athlete development. Considering the importance of this sub-stage, we would even propose that any sports coach, working with larger groups of athletes and aiming at skill learning, adheres to “Team-based Training” approaches and constantly introduces playing forms to training. In individualized “specialist coaching” constrained to small groups of athletes (which forms a focus of this paper), however, the skill training “sub-stages” of “Movement Variability Training” and “Complex Training” provide valuable alternatives. The authors would also like to acknowledge the challenge of a “specialist coach” working in isolation from team trainings and thus, encourage strong communication with the head coach or head of programme to ensure that long-term development of individual athletes is considered from an holistic and athlete-centered point of view. In any professional club or team set-up, there is a need for multi-disciplinary overview of development for each performer, so as to monitor progressions and avoid overtraining, injury or under-training; this overview is often the role of the head coach.

#### “Performance Training”

The third developmental stage of the “PoST” framework (Figure 1, the crimson-colored training section on the right in the framework) indicates a shift away from Newell's initial third stage of “skill optimization,” which originally aims to enhance the energy efficiency and adaptability of movements in perturbing and complex environments (Newell, 1996). According to the “PoST” framework, athletes in the “performance training” stage find themselves close to competition. Consequently, skill development may not necessarily be the primary focus (Farrow and Robertson, 2017), but rather exploiting the performance environment for maximum return or efficiency.

Particularly, on the one hand, “performance training” leading up to competition in team ball sports may initially contain training designs high in game-representativeness (i.e., perceived task complexity works closely around the red line in the days/weeks prior to competition). Under the overarching focus of optimizing team performances, soccer coaches, for example, would highlight performance-driven preparation in a team-tactical 11-vs.-11 training game. By explicitly instructing the “B-team” to replicate behavior of the upcoming opponent (e.g., formation or style-of-play), the training environment and individual exploration of movement opportunities is constrained by the coach; this is despite the training game itself being highly representative in terms of perceptual-cognitive and skill demands. On the other hand, closely preceding competition, factors such as performance stability and preparation through implementing athlete-led training routines (e.g., a pre-game warm-up routine led by the soccer GK) may be deemed (significantly) more important to athletes than learning and skill development. Therefore, it is proposed that the (specialist) coach in the “performance training” phase directly prior to competition re-highlights the importance of movement stability in order to build up confidence (i.e., perceived task complexity and game-representativeness stay well below the red solid line).

## PART C. Application of the “PoST” Framework to Training Planning

The former parts (A and B) have focused on the introduction of the “PoST” framework's theoretical foundation, underlying structure and the challenges that coaches need to consider when managing the training environment. In a final step, it is salient to demonstrate the framework's distinctive applied value for “specialist coaches” when used for practical training periodization and planning. Therefore, this section is particularly focused on: (1) the course of (multiple) training months and weeks (i.e., here described as macro- and micro-cycles); and (2) the structure of single training sessions (i.e., including single units within a training session). Notably, the following sub-sections will continue to use the soccer GK context; however, the authors encourage “specialist coaches” from other team ball sports to adjust and utilize the same conceptual structure for skill training periodization and planning.

### Application to Training Planning Over Macro- and Micro-Cycles

**Figure 7:**
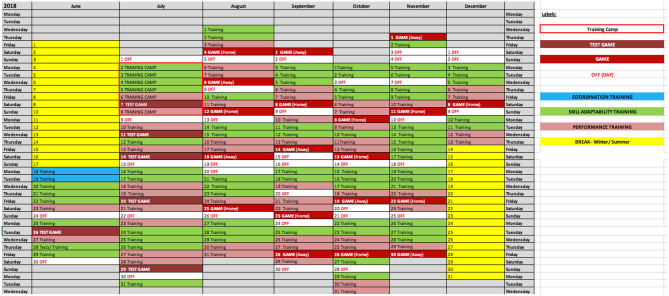
An example of a GK skill training periodization macro-cycle over the course of seven months during a professional soccer season.

**Figure 8:**
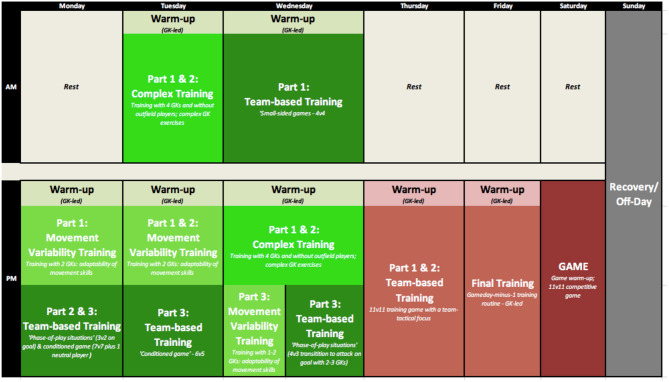
An example of the skill training (periodization) schedule of advanced (professional) U-20s youth GKs throughout a training week. Notably, the choice of training “sub-stages” and training content is constrained (1) by the number of GKs participating in the training session, and (2) by fitting the session around team-based training parts.

On the macro- and micro-levels for training planning, coaches may be confronted with the various aforementioned challenges; for example, taking into account the game-representativeness of the training task, the level of perceived task complexity and the athlete's skill development stage. In addition, further external variables, like the number of athletes involved in the training session, the competition schedule, and training focus (i.e., from technical-tactical, physical and psychological perspectives) constrain how skill training over multiple months, week, and sessions could be methodically structured. In order to provide practical examples, skill training periodization (based on the “PoST” framework) over the course of multiple months and weeks within a year-long season are presented. Figures 7, 8, here, display the training schedule of advanced under-20s youth GKs.

Firstly, the training periodization schedule in Figure 7 presents an example of training session planning over the course of seven months during a professional soccer season (i.e., a macro-cycle). In consideration of the proposed skill training stages (i.e., “Coordination Training,” “Skill Adaptability Training,” and “Performance Training”), the schedule includes various training and game activities, such as test games, competitive games, training camps, and regular training sessions. By systematically planning skill training months in advance, “specialist coaches” have the opportunity to gain an early insight into critical training weeks for skill learning (e.g., in contrast to training weeks with a focus on performance exploitation).

Secondly, Figure 8 exhibits an exemplary training week including various skill training sub-stages (i.e., a micro-cycle). In detail, the plan includes seven training sessions and a competitive game. The training sessions are periodized and planned based on the “PoST” framework's training stages of “Skill Adaptability Training” and “Performance Training”; for example, learning-focused “Movement Variability Training,” “Complex Training,” and “Team-based Training” during the first days of the week (i.e., indicated by the green-shaded boxes in Figure 8) is accompanied by training days focusing on “Performance Training” in preparation for competitive games (i.e., indicated by the maroon-shaded boxes). Particularly, this weekly pre-planned training schedule proves beneficial for “specialist coaches” when designing single training sessions in detail (see below).

Finally, we acknowledge that while the majority of “specialist coaches” may not have the luxury of periodizing skill training longitudinally, the framework itself has the scope to assist practitioners in this matter. Additionally, the “SAP” framework (see Farrow and Robertson, 2017) provides valid measures to monitor skill development.

### Application to Training Planning for Single Training Sessions

**Figure 9:**
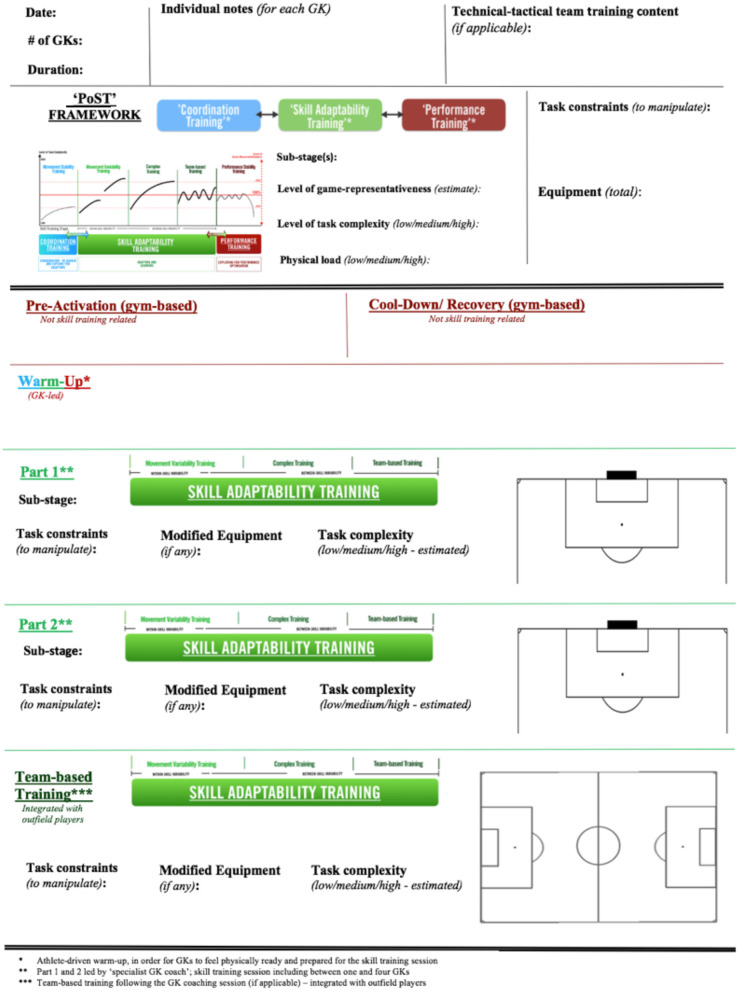
Example single training session template using the “PoST” framework to plan single units within a soccer GK training session. For this session plan, particularly, the stage of “Skill Adaptability Training” is the focus. Throughout part 1, part 2, and a “Team-based Training” part (if applicable), the coach can design a session based on: (1) task constraint manipulations; (2) equipment modification; and (3) estimated perceived task complexity for GKs involved in the session.

For training planning, the “PoST” framework offers an effective tool to guide coaches in the design of the structure of training sessions (including its various units). Prior to designing training exercises, the coach has the opportunity to pre-plan and individualize the methodical training approach. By selecting (1) relevant skill training (sub-) stages; (2) task constraints (that may be manipulated); (3) modifications of equipment (if applicable); and (4) estimating perceived task complexity and physiological load that is placed upon athletes, the coach can gain in-depth insight into the goals of the training session; this is before spending any time designing practical training exercises and games. For example, prior to a soccer GK training session, the “PoST” framework may provide a tool for designing and planning units within this single training session. Figure 9, hereof, displays a training session template specifically tailored toward the soccer GK training context.

Using the aforementioned example of advanced U-20s youth GKs, the structure of a Wednesday afternoon training session with four GKs could primarily focus on skill learning (see Figure 8). In detail, the session could commence with a brief and GK-led “warm-up” in order to physically and mentally prepare the GK for the session. Next, the session would quickly proceed toward “Complex Training.” Here, in particular, the coach could design a training environment that focuses on “between-skill variability.” Mainly limited by a small training group size of four GKs, training session parts 1 and 2 would aim to manipulate task constraints and equipment in order to develop the GK's ability to explore a complexity of affordances within the dynamic training environment. While training exercises at first are likely to be rather low in game-representativeness, they may be high in task complexity (i.e., due to modification of equipment and task constraints). Finally, toward the last part of this training session, the GK training group would be split depending on the coach's observations in parts 1 and 2 (e.g., a GK that was overly challenged by task complexity would be separated from less challenged GKs in part 3). For example, one GK would move into “Movement Variability Training” to focus on “within-skill variability,” rather low in task complexity (i.e., adapting movement parameters in response to changing constraints). Simultaneously, the other GKs would join the outfield players for “phase-of-play situations,” which would be rather high in task complexity (e.g., more game-representative 4v3 transitions to attack on goal).

Overall, “specialist coaching” commonly displays constraints such as working with limited athlete numbers or having to fit training session parts around team-based training (see example above). Thus, it remains an important challenge for these “specialist coaches” to enforce non-linearity throughout single session designs (e.g., similar to training designs applied by alternative approaches, such as GBAs).

## Concluding Remarks

In summary, this paper pursues the goal of practically applying skill training theory by proposing a skill training periodization framework. The “PoST” framework has the potential to provide guidance for “specialist sport coaches” (not only in soccer goalkeeping) on designing appropriate training environments that consider the representative demands of competition. In particular, three aspects appear to underline the framework's merit: (1) the applicability to training planning both on the macro- and micro-level (i.e., over the course of multiple training months and weeks); (2) the applicability to single training session and unit planning; and (3) the support for practitioners to adequately manipulate the training environment toward individual athletes' needs; i.e., by introducing training challenges under consideration of representative game demands and the athlete's perceived level of task complexity.

Despite the proposed framework providing valuable practical support for sport coaches, its theoretical limitations need to be addressed. These limitations aim to encourage future multi-disciplinary research based on the proposed skill training stages. Firstly, the paper does not provide quantitative approaches toward assessing game-representativeness of training; however, the “SAP” framework by Farrow and Robertson (2017) does an excellent job of this. By focusing on comprehensively transferring academic skill training theory to practical coaching, the “PoST” framework proposes estimates of game-representativeness for certain skill training stages. The validation of “practice assessment tools,” such as a recently introduced tennis-specific assessment tool would add further merit (see Krause et al., 2018). Additionally, the measurement of action fidelity and functionality may prove valuable in assessing game-representativeness of training (Farrow and Robertson, 2017; Krause et al., 2019). Particularly, the use of technological tools applied in performance analysis (e.g., wearable technologies or video analysis software) may assist researchers and coaches in comparing training designs with performance environments; for example, Travassos et al. (2012) measured action fidelity and training task representativeness by comparing ball speed and passing accuracy in various futsal training conditions with data from competitive games. Secondly, due to its qualitative nature, the concept of information complexity makes it difficult for coaches to evaluate what athletes may perceive as (too) complex. Consequently, the “PoST” framework proposes the use of a subjective concept for individualizing skill training; this approach needs further exploration and empirical research in order to scale and objectively measure informational complexity as perceived by athletes during skill training. For example, researchers could apply and refine internal measurement protocols for monitoring athletes' perceived informational complexity, such as the recently introduced “rating of perceived challenge” (RPC; see Hendricks et al., 2019). These internal evaluations could be further combined with external measures, such as the actual output produced by an athlete (e.g., the passing accuracy of a soccer player; e.g., Chow et al., 2008). On this subject, a more complex training task “is assumed as a proxy for increased error” (Farrow and Robertson, 2017, p. 1047).

As a final remark, understanding that there is “no silver bullet for all teaching (coaching) and learning” represents the responsibility for coaches to individualize and periodize skill training based on athletes' needs (Renshaw et al., 2010, p. 135). Considering the emerging context of “specialist coaches” working solely with individual athletes or small groups of athletes, there appears to be a fruitful opportunity for individualized control of information complexity that athletes are confronted with. If a coach working individually with one or two athletes cannot manipulate task constraints in order to cater to each individual athlete's needs, then one might argue that this role would be even harder for a head coach working with a squad of 20–30 athletes at the same time.

## Ethics Statement

Written informed consent was obtained from the individual(s), and minor(s)' legal guardian/next of kin, for the publication of any potentially identifiable images or data included in this article.

## Author Contributions

FO developed the conception of the model. S-KM and SK contributed to re-design and presentation of the model. FO wrote the first draft of the manuscript. S-KM and SK wrote sections of the manuscript. All authors contributed to manuscript revision, read, and approved the submitted version.

### Conflict of Interest

The authors declare that the research was conducted in the absence of any commercial or financial relationships that could be construed as a potential conflict of interest.
